# An intraventricular meningioma and recurrent astrocytoma collision tumor: a case report and literature review

**DOI:** 10.1186/s12957-015-0436-6

**Published:** 2015-02-12

**Authors:** Duoduo Zhang, Jinlu Yu, Yunbao Guo, Shujie Zhao, Guoguang Shao, Haiyan Huang

**Affiliations:** Department of Surgery, First Hospital of Jilin University, 71 Xinmin Avenue, Changchun, 130021 China; Department of Neurosurgery, First Hospital of Jilin University, 71 Xinmin Avenue, Changchun, 130021 China; Department of Intensive Care Unit, First Hospital of Jilin University, 71 Xinmin Avenue, Changchun, 130021 China

## Abstract

**Background:**

Intracranial meningioma and glioma collision tumors are relatively uncommon and are even more rarely located within the ventricles.

**Case presentation:**

Here, we report a case of a patient with an intraventricular meningioma and astrocytoma collision tumor. A 39-year-old man previously underwent excision of an astrocytoma in the triangle area of the lateral ventricle and exhibited good post-surgery recovery. The astrocytoma recurred *in situ* six years after the surgery, and the case was complicated by a malignant meningioma. The patient recovered well after surgery to treat the recurrence and was administered radiotherapy after discharge. In addition to reporting on this case, we conducted a literature review of collision tumors; based on this review, we propose several hypotheses regarding the formation of collision tumors.

**Conclusions:**

We conclude that a possible cause of the collision tumor formation between the intracranial meningioma and the astrocytoma was the recurrence of an astrocytoma-induced malignancy of the arachnoid cells in the choroid plexus.

## Background

An intracranial collision tumor represents two coexisting, histologically distinct primary tumors in the same brain region. This type of tumor involves a collision between meningiomas and other intracranial tumors, such as gliomas, pituitary adenomas and schwannomas, and is clinically uncommon [1,2]. The most common type is a collision tumor comprising a meningioma and a glioma [3-5]. Other collision tumor types include those comprising meningioma and pituitary adenoma, meningioma and schwannoma, metastasis and schwannoma, and craniopharyngioma and pituitary adenoma [3,5-7]. It has been previously reported that the base of a meningioma is mostly attached to the cerebral falx, the convexity of the dura mater, and the base of the skull, and gliomas are usually distributed at the periphery of meningiomas. The two tumor types can occur simultaneously, or a glioma can appear after the treatment of a meningioma [5,8-10]. However, meningioma very rarely occurs concurrently with recurrent glioma following surgical excision of the original glioma from the lateral ventricle, and such coincidences are usually related to radiotherapy [11-13]. The meningioma in a collision tumor reported here not only occurred after the excision of an astrocytoma without radiotherapy but was also located in the middle of the astrocytoma. Furthermore, the collision tumor occurred within the lateral ventricle, which is very rare. We have not found any such cases reported in the existing literature.

## Case presentation

**Figure 1:**
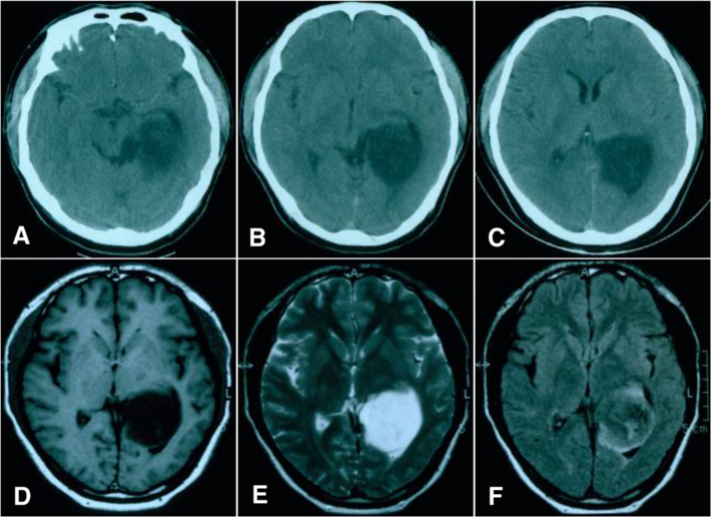
**Computed tomography (CT) and magnetic resonance imaging (MRI) images of an astrocytoma recorded in 2004. (A-C)** Head CT shows a lesion that appears as a low-density region in the left lateral ventricle trigone that is exerting pressure on the surrounding tissues; the central structure remains visible in the middle of the brain. **(D-F)** MRI imaging showing low signaling on T1WI and high signaling T2WI and FLAIR imaging showing slightly higher signaling; the lesion was located at the trigone.

**Figure 2:**
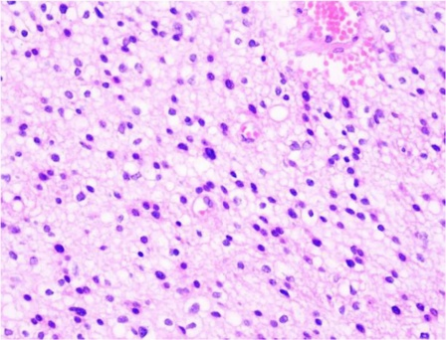
**Pathological examination after surgery (hematoxylin and eosin (H&E) × 400).** The tumor comprised multiangular cells with highly dense and slightly atypical nuclei. Astrocytoma was diagnosed as World Health Organization (WHO) class II.

**Figure 3:**
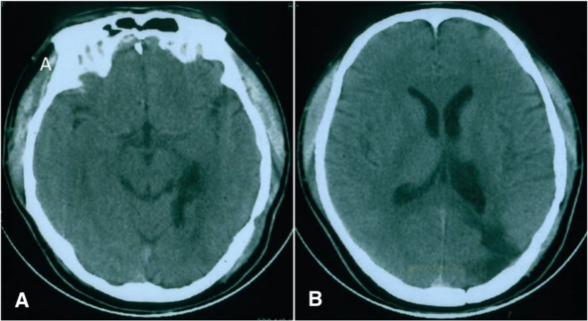
**Postoperative computed tomography (CT) examination in 2005.** **(A-B)** No recurrence of astrocytoma was found.

**Figure 4:**
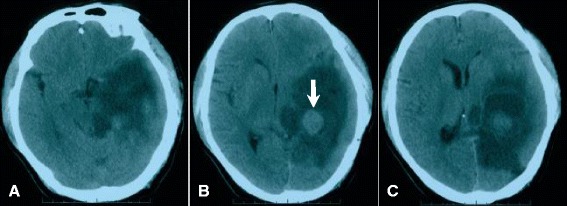
**Computed tomography (CT) examination showing astrocytoma recurrence in October 2010. (A-C)** A low-density lesion was located in the left lateral ventricle trigone and appeared irregular and cystic; a round, high-density lesion was observed in the center of the cystic tumor (arrowed in B).

**Figure 5:**
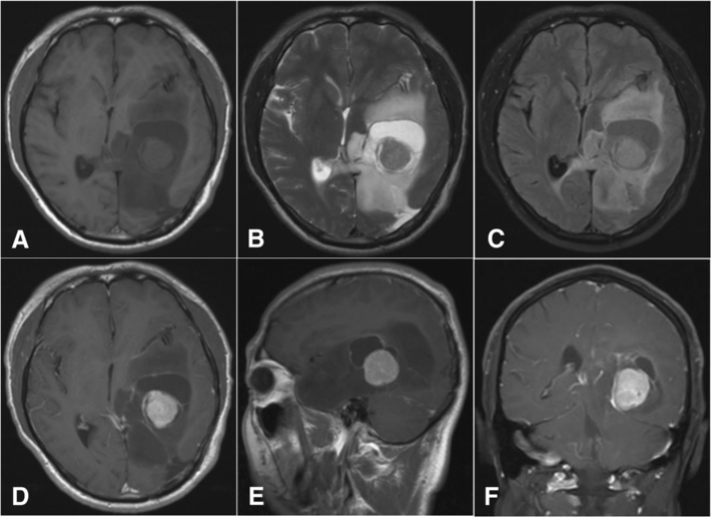
**Magnetic resonance imaging (MRI) images recorded in October 2010.** Head MRI scan **(A-C)** and enhanced MRI **(D-F)** images showing the enhancement on the cystic tumor wall and the lesion in the center of the cystic tumor.

**Figure 6:**
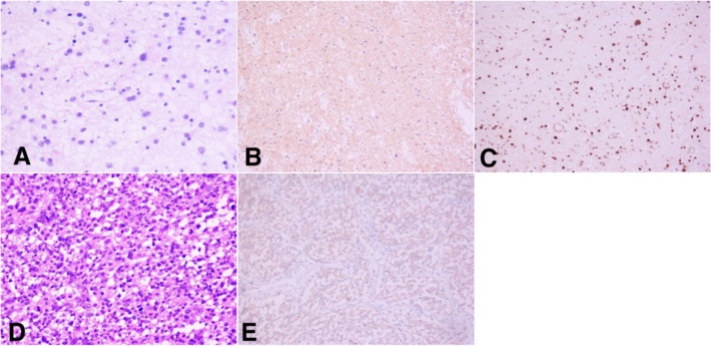
**Hematoxylin and eosin (H&E) staining showing the results of pathological examinations. (A-C)** Oligodendrocytes-astrocytoma (World Health Organization (WHO) class II). **A** The tumor comprised small branch glioma and astrocytes (HE × 200). **(B)** Positive immunohistochemical staining for gilial fibrillary acidic protein (GAFP) (Envision method × 200). **(C)** Positive immunohistochemical staining for oligodendrocyte lineage transcription factors (Oligo) (Envision method × 200). **(D-E)** Malignant meningioma. **(D)** Several tumor cells were distributed extensively and appear obviously atypical and mitotic (H&E × 200). **(E)** Immunohistochemical staining showing epithelial membrane antigen (EMA) (Envision method × 200).

The patient was a 39-year-old man. Computed tomography (CT) and magnetic resonance imaging (MRI) examinations were performed in January, 2004 to diagnose headaches. The result revealed an intraventricular lesion, which was cystic and located in the left lateral ventricle trigone; no edema in the periphery of the lesion was observed (Figure 1). After the tumor was excised, a pathological examination was performed. The results confirmed that the tumor was a glioma (astrocytoma World Health Organization (WHO) class II) (Figure 2). The patient recovered well after surgery, and no complications developed; the patient did not receive radiotherapy. In 2005, MRI reexamination did not show recurrence of the astrocytoma (Figure 3). In October 2010, the patient complained of headaches and dizziness. A CT examination revealed recurrence of the astrocytoma in the left lateral ventricle trigone, and a large area of edema was observed in the periphery of the temporal-parietal-occipital lobe. The tumor was cystic and exhibited a round, high-density area in the center (Figure 4). Using a head MRI scan and enhanced MRI examination, not only was enhancement observed in the wall of the cystic tumor but stronger enhancement was observed in the center of the tumor (Figure 5); the rest of the lesion exhibited the same intensity as normal tissue. Physical examination revealed almost no neurological signs except bilateral papilledema. Astrocytoma was diagnosed based on the imaging results and the clinical history. Craniotomy was performed to confirm the pathological changes in the tumor. The surgery was performed via the left temporal-occipital lobe. The cavity of the tumor was pierced during the operation, and five mL of yellow liquid was withdrawn. The cystic part of the tumor was then exposed after opening the surface of the tumor. This part was soft and appeared collagen-like; the blood supply was minimal and was removed with suction. The center of the lesion was exposed after removal of the cystic part at the periphery of the astrocytoma; the lesion center was hard, had an extremely abundant blood supply, and exhibited a clear boundary. The base of the lesion was tightly attached to the choroid plexus in the lateral ventricle. The lesion was excised under a microscope; then, the cystic wall of the lesion, which did not have a clear boundary with the surrounding brain tissues, was removed. Pathological examination after surgery confirmed that the cystic part of the tumor was a mixed glioma (oligodendrocytes-astrocytoma WHO class II) that tested positive for glial fibrillary acidic protein (GFAP) and oligodendrocyte lineage transcription factors (Oligo. The center of the lesion was a malignant meningioma (WHO class II) that exhibited 80% positive staining for both Ki-67 and epithelial membrane antigen (EMA). The two tumor types did not invade each other (Figure 6). The patient recovered well but developed bilateral homonymous hemianopia post surgery. He received gamma-knife radiotherapy after discharge. Tumor recurrence did not occur during a one-year follow-up.

### Discussion

As mentioned previously, no clear explanation is found in the literature regarding the cause of the collision between intracranial meningioma and glioma, and few reports describe this condition. Nagashima *et al*. reported one case of a sphenoid ridge meningioma and cystic astrocytoma collision tumor in 1963. They also reviewed the literature regarding the coexistence of intracranial meningioma and glioma since 1938 and found several cases of collision tumor [14]. Strong *et al*. reported two cases of a meningioma and cystic astrocytoma collision tumor in 1976 [15]. Since then, further collision tumors have been reported. In 1991, Spallone *et al*. reviewed 57 cases of coexisting meningioma and glioma and found that 18 of these cases were collision tumors [16]. Other cases have been reported as follows: one by Prayson *et al*. in 2002 [17]; one by Drlicek *et al*. in 2004 [18]; three by Nestler *et al*. in 2007 [19]; one by Mitsos *et al*. in 2009 [20]; and one by Chen *et al*. in 2010 [21]. Nevertheless, our understanding of collision tumors comprising meningioma and glioma remains limited. Here, we report a case involving an intracranial collision tumor that was located in the lateral ventricle; moreover, the meningioma was located in the center of the glioma, which is different from the previously reported cases.

The intracranial meningioma and astrocytoma collision tumor reported here is very rare. This collision tumor exhibited several unique features: (1) the tumor was located within the ventricle; (2) the meningioma was located in the center of the astrocytoma; (3) the original astrocytoma occurred earlier than the meningioma: when the astrocytoma reoccurred post surgery, the meningioma developed simultaneously; (4) the coexistence of the tumors was not due to the induction of radiotherapy; (5) the meningioma was malignant. Based on our literature review, meningioma in a collision tumor is usually attached to the dura mater, and most meningiomas are located beside the cerebral falx, either on the convexity of the meningioma or on the base of the skull. When collision occurs between a meningioma and astrocytoma, the astrocytoma is usually distributed on the periphery of the meningioma [14-21]. Spallone *et al*. reported that among 18 cases of collision tumors, 16 were located on the convexity of a meningioma [16]. No case of a collision tumor within a ventricle has been reported. The collision tumor reported here was not only located within the ventricle but was located in the center of the ventricle.

The development of a meningioma and astrocytoma collision tumor is a dynamic process; one type of tumor occurs earlier, and the second type of tumor occurs later after in response to certain factors. However, it is difficult to observe the dynamic process during follow-up. Mitsos *et al*. reported one case of glioma recurrence after surgery for sphenoid ridge meningioma [20]. Pereira *et al*. reported an atypical case of glioma occurrence six months after meningioma excision [22]. However, the dynamic development of a collision tumor cannot be monitored in most cases, because even imaging studies cannot facilitate a definite diagnosis after the patients present clinical symptoms, and only pathological examinations can confirm the diagnosis of a collision tumor [8-10,23]. Thus, monitoring the dynamic development of a collision tumor is a prerequisite for providing a reasonable explanation for the causes of the tumor. In the present study, we found the concurrence of the malignant meningioma six years after recurrence of the astrocytoma. To the best of our knowledge, this case represents the first successful observation of the occurrence and development of a collision tumor between malignant meningioma and glioma. Moreover, no radiotherapy and chemotherapy was administered after the excision of the original astrocytoma, and these processes are similar to the dynamic development of a collision tumor as described above.

The cause of the intracranial meningioma and glioma collision tumor remains unclear; however, several hypotheses are possible [21,24-28]. (1) The simple concurrence of the tumors might have occurred by chance because both intracranial meningioma and glioma are common types of brain tumors. (2) The collision tumor might have been induced by radiotherapy, trauma or surgical history. (3) A malignant tumor might have acted as an inducing factor for the generation of another type of a tumor, leading to the malignant transformation of surrounding brain tissues and arachnoid cells. (4) A glioma might have stimulated the growth of a meningioma. Vaquero *et al*. reported one case of a collision tumor between meningioma on the convexity of the brain and a glioma; pathologically, a transient area between the meningioma and astrocytoma was observed, and the two tumor types were mixed in some areas, supporting hypotheses (3) and (4) [9]. Drlicek *et al*. reported one case of the concurrence of a sphenoid ridge meningioma with a peripheral collision tumor. Pathological examination revealed that a meningioma was located within a glioma, also supporting hypotheses (3) and (4) [18]. Prayson *et al*. reported one case of a collision tumor comprising a meningioma in the sagittal sinus in the frontal lobe and a peripheral glioma; pathologically, the two tumors invaded each other, supporting hypotheses (3) and (4) [17].

Even if the two tumor types are adjacent to each other, they might not invade each other. In 2007, Nestler *et al*. reported three cases of meningioma and glioma concurrence, one of which was a collision tumor. Although the two tumor types were adjacent, pathological examination did not show invasion between the tumors. Therefore, we think this collision tumor formed by chance, supporting hypothesis (1) [19].

Davis *et al*. reported three cases of meningioma and glioma concurrence; a detailed discussion of the causes of this type of collision tumor was provided, but no pathological invasion was observed. This case supports hypotheses (3) and (4). Although one of these three cases had an obvious history of trauma, the observed collision tumor occurred 30 years after the trauma [25]. Few cases of meningioma after trauma have been reported [7,29]. The occurrence of glioma has been reported after trauma [30,31]; therefore, it appears that trauma might play a role in the formation of collision tumors. Radiotherapy has also been implicated in glioma development, because irradiation-induced intracranial meningioma and glioma have been reported. Pereira *et al*. reported that one case of nontypical meningioma had received radiotherapy after surgical excision, and glioma developed *in situ* six months after the surgery [22]. Zuccarello *et al*. reported one case of glioblastoma that was found 10 years after the excision and radiotherapy of an intracranial meningioma [27]. Campbell *et al*. reported that multiple meningiomas occurred eight years after the excision and radiotherapy of medulloblastoma [32]. Martínez-Lage *et al*. reported that a meningioma occurred four years after the excision and radiotherapy of an astrocytoma [33].

The meningioma and astrocytoma collision tumor reported here exhibited special features. This patient underwent surgical excision of an astrocytoma in the lateral ventricle but was not administered radiotherapy post surgery. The recurrence of astrocytoma occurred 10 years after follow-up, and the meningioma was found in the center of the astrocytoma. However, pathological examination did not show invasion, and a clear boundary was observed between the two tumor types. Moreover, the content and blood supply of the two tumor types were different. Based on these findings, we conclude that the astrocytoma occurred earlier and induced the formation of the meningioma, supporting hypotheses (3) and (4) described above. The astrocytoma induced the development of a malignant meningioma caused by the malignant transformation of arachnoid cells in the choroid plexus of the lateral ventricle. In addition, stimulation of the astrocytoma promoted the growth of the meningioma. Furthermore, a clear history of surgery might have contributed to the development of the meningioma.

## Conclusions

Collision tumors rarely comprise an intraventricular meningioma and an astrocytoma. We conclude that one possible cause of the development of such a collision tumor is the recurrence of an astrocytoma-induced malignancy of arachnoid cells in the choroid plexus. In addition, stimulation from an astrocytoma apparently induced the growth of the meningioma.

## Consent

Written informed consent regarding the publication of this case report and its accompanying images was obtained from the patient. Copies of the written consent are available for review upon request.
